# The microRNA-29ab1/Zfp36/AR Axis in the Hypothalamus Regulates Male-Typical Behaviors in Mice

**DOI:** 10.3390/ijms252313089

**Published:** 2024-12-05

**Authors:** Jie Ma, Yingying Lin, Wei Xiong, Xiaoxue Liu, Minghui Pan, Jiazeng Sun, Yanan Sun, Yixuan Li, Huiyuan Guo, Guofang Pang, Xiaoyu Wang, Fazheng Ren

**Affiliations:** 1 College of Food Science and Nutritional Engineering, China Agricultural University, Beijing 100083, China; majiefsat@163.com; 2 Key Laboratory of Precision Nutrition and Food Quality, Department of Nutrition and Health, China Agricultural University, Beijing 100193, China

**Keywords:** *miR-29ab1*, aggression, mating, hypothalamus, androgen receptor

## Abstract

Male-typical behaviors such as aggression and mating, which reflect sexual libido in male mice, are regulated by the hypothalamus, a crucial part of the nervous system. Previous studies have demonstrated that microRNAs (miRNAs), especially *miR-29*, play a vital role in reproduction and the neural control of behaviors. However, it remains unclear whether *miR-29* affects reproduction through the hypothalamus-mediated regulation of male-typical behaviors. Here, we constructed two mouse knockout models by ablating either the *miR-29ab1* or *miR-29b2c* cluster. Compared to WT, the ablation of *miR-29ab1* in male mice significantly reduced the incidence of aggression by 60% and the incidence of mating by 46.15%. Furthermore, the loss of *miR-29ab1* in male mice led to the downregulation of androgen receptor (AR) in the ventromedial hypothalamus. Transcriptomic analysis of the hypothalamus of *miR-29ab1*-deficient mice revealed inflammatory activation and aberrant expression of genes associated with male-typical behaviors, including *Ar*, *Pgr*, *Htr4*, and *Htr2c*. Using bioinformatics analysis and dual-luciferase reporter assays, we identified zinc finger protein 36 (*Zfp36*) as a direct downstream target gene of *miR-29ab1*. We subsequently showed that ZFP36 colocalized with AR in GT1-7 cells. Furthermore, inhibition of *Zfp36* or *RelB* in GT1-7 cells led to an increase in AR expression. Collectively, our results demonstrate that the *miR-29ab1/Zfp36/AR* axis in the hypothalamus plays a pivotal role in the regulation of aggression and mating in male mice, providing a potential therapeutic target for treating infertility caused by low libido.

## 1. Introduction

Infertility affects 186 million people worldwide, with more than half of all cases due to male-associated factors [1,2,3]. Most studies on male infertility focus on sex hormones, spermatogenesis, and testicular health [4,5]. However, men can also experience difficulties associated with sexual interest and desire [6,7]. Studies have reported that 4.7–27.9% of men from various countries have experienced hypoactive sexual desire [8,9]. Aging, chronic diseases, and hormonal dysregulation have been shown to lead to a decrease in sexual desire [5,9,10]. However, the mechanisms of low libido remain poorly understood due to the complexity of the human body and limitations of human research.

Mouse models offer an excellent avenue for understanding the neural control of sexual desire. Typically, male mice exhibit male-typical behaviors when caged with mature conspecifics of the opposite sex, including aggression and mating, to demonstrate their rejection or desire for the opposite sex [11,12,13]. Male-typical behaviors are mainly regulated by testosterone in adult male mice, which activates the neural circuits of male-typical behaviors by binding to androgen receptor (AR) [12,14,15]. Mice that are either castrated or genetically modified to lack AR do not exhibit male-typical behaviors [16,17]. Further studies using conditional knockout mouse models have shown that early deletion of AR in neuronal cells impaired male-typical behaviors, without affecting their peripheral functions [15]. Notably, the hypothalamus has been identified as the most critical site of male-typical behaviors based on electrical stimulation and genetic inhibition experiments [11,18,19]. On the one hand, the hypothalamus is the most prominent downstream target of other brain regions related to male-typical behaviors, such as the medial and cortical amygdaloid areas. On the other hand, the ventromedial hypothalamus (VMH), which is known to regulate male-typical behaviors and contains a high concentration of AR [11,20,21]. Therefore, the homeostatic expression of AR in the hypothalamus is essential for the manifestation of male-typical behaviors.

In the last decade, microRNAs (miRNAs), a category of noncoding RNAs, have been identified as crucial modulators of AR signaling at the post-transcriptional level, as they bind to the 3′-UTRs of target mRNAs [22,23,24]. A single miRNA can target hundreds of different mRNAs, underscoring their expansive role in regulating gene expression [25]. Recent studies have shown an association between miRNAs and neurobehavioral modulation [24,26,27]. However, whether miRNAs regulate male-typical behaviors by affecting AR remains unknown. Several studies have shown that the *miR-29* family, broadly expressed in the nervous system, is associated with the regulation of animal behaviors [28,29,30]. The three components of *miR-29* family, namely *miR-29a*, *-29b*, and *-29c*, possess an identical seed sequence. Two gene clusters located on chromosome 6 and chromosome 1 are transcribed as *miR-29ab1* and *miR-29b2c*, respectively [31]. While *miR-29b1* and *miR-29b2* share identical sequences, *miR-29a* and *miR-29c* differ by a single nucleotide [32]. Notably, mice deficient in the *miR-29ab1* cluster exhibit sterility [33]. In females, *miR-29ab1*-deficient mice showed normal egg development but displayed ovulation disorders and impaired luteinizing hormone secretion [34]. Hypothalamic-specific inhibition of *miR-29* targeting *Tbx21* directly stimulates *Gnrh1* expression, leading to earlier puberty in prepubertal females [35]. Interestingly, testis and seminal vesicle weights were found to be normal in *miR-29ab1* mutant male mice [34], while brain-specific knockdown of *miR-29* led to decreased fertility in mice [36]. However, whether *miR-29* affects reproduction by regulating male-typical behaviors remains unknown.

Here, we found that male *miR-29ab1*-deficient mice were infertile, and that this infertility may be due to impaired male-typical behavior. Remarkably, alterations in male-typical behaviors in *miR-29ab1*-deficient mice were associated with the downregulation of the AR in the hypothalamus. We further identified zinc finger protein 36 (*Zfp36*) as a target gene, which modulates AR expression through RelB. Collectively, our results identify a regulatory role of *miR-29ab1* in the neurobehavioral control of reproduction, which may be beneficial for understanding infertility resulting from hypoactive sexual desire in humans.

## 2. Results

### 2.1. Genetic Ablation of miR-29ab1 Results in the Loss of Male-Typical Behaviors

**Figure 1:**
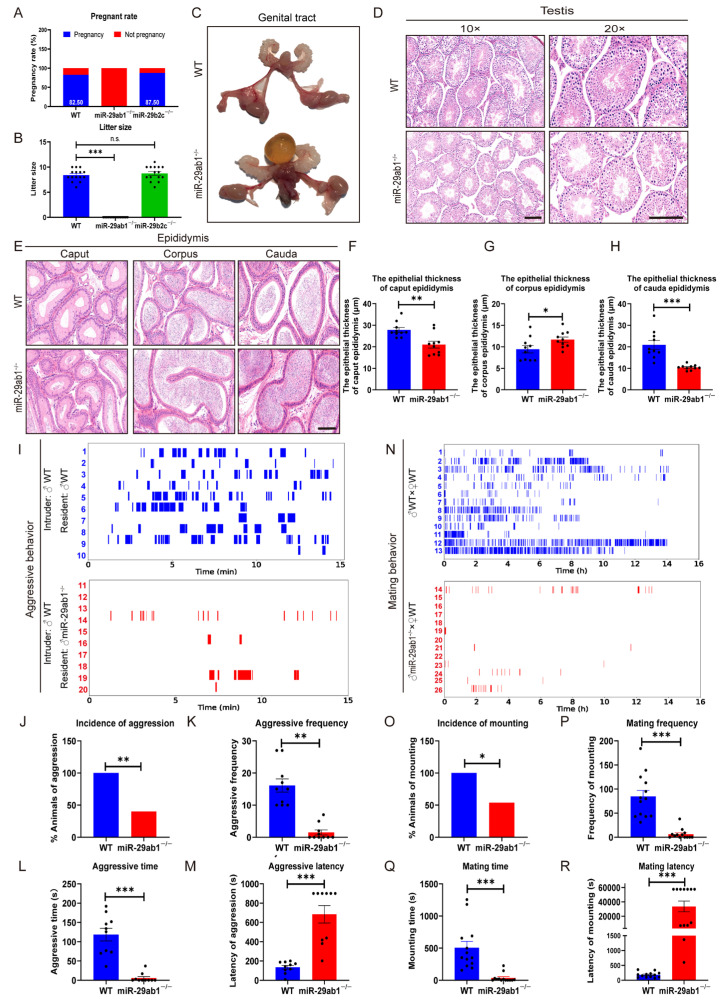
The ablation of *miR-29ab1* leads to infertility, which may be due to impaired aggressive and mating behaviors in male mice. (**A**) Pregnancy rate (*n* = 10). (**B**) Litter size (*n* = 15). (**C**) Representative images showing the genital tract of reproductive tissues in male mice. (**D**,**E**) H&E staining of the testis (**D**) and epididymis (**E**). (**F**–**H**) The epithelial thickness of the caput (**F**), corpus (**G**), and cauda epididymis (**H**) (*n* = 10). (**I**) Raster plots showing the individual aggression of WT and *miR-29ab1*^−/−^ mice towards an intruder of WT male during the aggressive behavior test (15 min). Blue numerals 1–10 denote that the resident mice are male WT mice, while red numerals 11–20 indicate that the resident mice are male *miR-29ab1^−/−^* mice. Each vertical line represents an attack by the resident male mouse on the intruder male mouse (*n* = 10). (**J**–**M**) Quantitative analysis of aggressive animal behavior. Percentage of animals displaying aggressive behavior (*n* = 10) (**J**). Frequency of aggressive behavior (*n* = 10) (**K**). Amount of time displaying aggressive behavior (*n* = 10) (**L**). Latency to aggressive behavior. Each dot represents a mouse (*n* = 10) (**M**). (**N**) Raster plots showing the individual mounting of WT and *miR-29ab1*^−/−^ mice towards female WT mice during the mating behavior test (15 h). Blue numerals 1–13 denote the male WT mice, while red numerals 14–26 indicate the male *miR-29ab1^−/−^* mice. Each vertical line represents a mounting attempt by the male mouse on the female mouse (*n* = 13). (**O**–**R**) Quantitative analysis of mating behavior in animals. Percentage of animals displaying mounting (*n* = 13) (**O**). Frequency of mating behavior (*n* = 13) (**P**). Time of mating (*n* = 13) (**Q**). Latency to mating behavior. Each dot represents a mouse (*n* = 13) (**R**). Scale bars, 100 μm; * *p* < 0.05, ** *p* < 0.01, *** *p* < 0.001, n.s., not significant.

*MiR-29*-null mice (*miR-29*^−/−^ mice) were produced by breeding *miR-29ab1*^+/−^ and *miR-29b2c*^−/−^ mice. However, since *miR-29*^−/−^ mice perished 30 days post-birth, we subsequently used *miR-29ab1*^−/−^ or *miR-29b2c*^−/−^ mice to evaluate adult male reproduction. The results showed that 82.5% of WT female mice were successfully fertilized by WT male mice, while 87.5% achieved fertilization when paired with *miR-29b2c*^−/−^ male mice. However, WT females mated with *miR-29ab1*^−/−^ male mice produced no pregnancies (Figure 1A). Meanwhile, the litter sizes from *miR-29b2c*^−/−^ males were comparable to those of WT males (Figure 1B).

Detailed morphological examination of genitalia revealed significant bladder distension in *miR-29ab1*^−/−^ mice relative to WT mice, whereas the genital tract of *miR-29ab1*^−/−^ mice appeared comparable to that of WT mice (Figure 1C). Quantitative analyses indicated that there were no significant disparities in the testicular or epididymal coefficients between WT and *miR-29ab1*^−/−^ mice (Figure S1A,B). Moreover, histological assessments of the testis and epididymis revealed no structural abnormalities between WT and *miR-29ab1*^−/−^ mice (Figure 1D,E). However, H&E staining revealed that the epithelial thickness of the caput epididymis (−24.56%) and cauda epididymis (−50.07%) was significantly reduced in *miR-29ab1*^−/−^ mice relative to WT mice, while the epithelial thickness of the corpus epididymis (+23.74%) was increased (Figure 1F–H). Sperm motility and morphology were similar between WT and *miR-29ab1*^−/−^ mice (Table 1). Subsequently, an in vitro fertilization assay confirmed that *miR-29ab1*^−/−^ sperm were able to fertilize eggs, leading to viable offspring, indicating that the observed infertility is not due to inherent abnormalities in the testis, epididymis, or sperm but may involve other factors that remain unidentified. 

Aggressive and mating behaviors are essential for the completion of reproduction. First, both WT and *miR-29ab1*^−/−^ mice displayed peripheral and central activity (Figure S2A), as well as similar locomotor activity times and distances (Figure S2B,C). Furthermore, the total fecal boli count of *miR-29ab1*^−/−^ mice was similar to that of WT mice (Figure S2D). These findings indicate that the absence of *miR-29ab1* does not trigger abnormal spontaneous activity or anxiety in male mice. 

Next, WT resident males typically attacked intruder males, while a significant reduction in aggressive behavior toward intruders was observed in *miR-29ab1*^−/−^ mice (40%, 4/10) compared to WT mice (100%, 10/10) in the resident-intruder test (Figure 1I,J), with significantly reduced aggressive frequency (−0.91-fold) and time (−7.00-fold) (Figure 1K,L), and increased latency to aggression (+34.05-fold) (Figure 1M). 

During the mating test, all WT males displayed mounting attempts, while only 53.85% of *miR-29ab1*^−/−^ mice displayed mounting behavior (Figure 1N,O). The frequency (−0.94-fold) and duration (−0.97-fold) of mounting were significantly decreased in *miR-29ab1*^−/−^ mice (Figure 1O–Q), while the latency to first mount was 285-fold longer for *miR-29ab1*^−/−^ mice than WT male mice (Figure 1R). Notably, the aggressive behavior of *miR-29b2c*^−/−^ male mice was also reduced (Figure S3A), while a high frequency of mating behavior can be observed in *miR-29b2c*^−/−^ male mice (Figure S3B). These findings indicate that a partial deficiency of *miR-29* results in sterility, underscoring the critical role of *miR-29ab1* in reproductive function. However, the cause of infertility in *miR-29ab1*^−/−^ mice may be at least in part due to impaired mating behavior.

### 2.2. Deletion of miR-29ab1 Decreases AR Expression in the Hypothalamus

**Figure 2:**
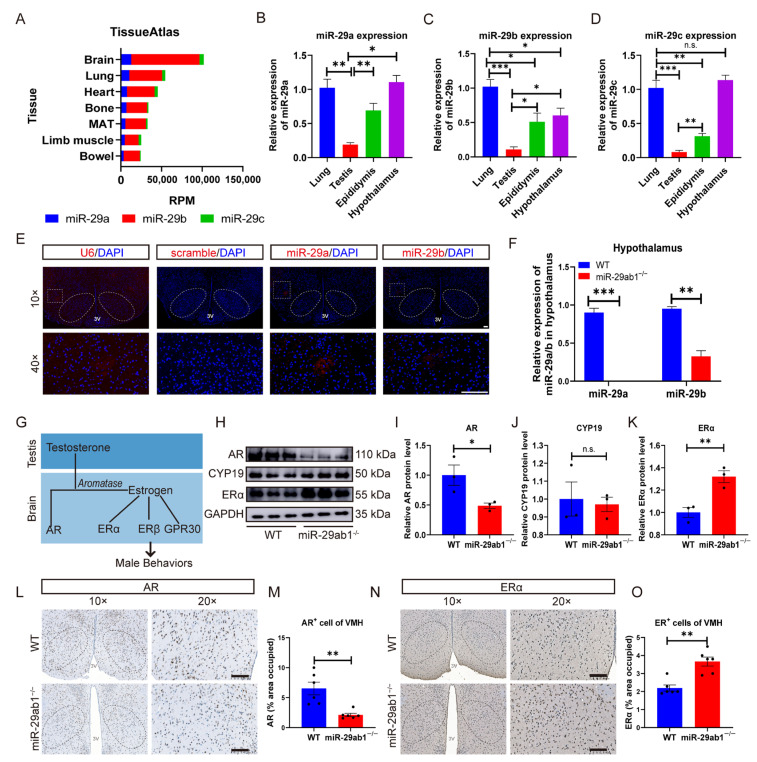
*MiR-29a* and *miR-29b* are enriched in the hypothalamus, and ablation of *miR-29ab1* impairs expression of AR in the hypothalamus. (**A**) *miR-29* expression profiles in adult mice calculated from the mean expression levels across seven tissues in the TissueAtlas database. (**B**–**D**) Relative levels of *miR-29a* (**B**), *miR-29b* (**C**), and *miR-29c* (**D**) in the lung, testis, epididymis, and hypothalamus of adult male WT mice (*n* = 6). (**E**) FISH analysis showing U6, scramble, *miR-29a*, *miR-29b* probes in the hypothalamus (VMH: white dashed circle). (**F**) Relative expression of *miR-29a* and *miR-29b* in the hypothalamus of WT and *miR-29ab1*^−/−^ mice (*n* = 6). (**G**) Schematic showing how the receptors of sex steroid hormones control male behaviors. (**H**) Representative western blot showing AR, CYP19, and ERα protein expression levels in the hypothalamus (*n* = 3). GAPDH served as the loading control. (**I**–**K**) Quantification of western blot data. Relative AR (**I**), CYP19 (**J**), and ERα (**K**) expression levels in the hypothalamus were normalized to GAPDH (*n* = 3). (**L**) Representative immunohistochemical image showing AR staining in the hypothalamus (VMH: black dashed circle). (**M**) The number of AR^+^ cells in the VMH of WT and *miR-29ab1*^−/−^ mice (*n* = 6). (**N**) Representative immunohistochemical image showing ERα staining in the hypothalamus (VMH: black dashed circle). (**O**) The number of ERα^+^ cells in the VMH of WT and *miR-29ab1*^−/−^ mice (*n* = 6). Scale bars, 100 μm; MAT, mesenteric adipose tissue, RPM, Reads Per Million; 3V, third ventricle; VMH, ventromedial hypothalamus; AR, androgen receptor; CYP19, Cytochrome P450 19; ERα, estrogen receptor alpha; DAPI, 4′6-diamidino-2-phenylindole. * *p* < 0.05, ** *p* < 0.01, *** *p* < 0.001, n.s., not significant.

Initial analysis of tissue expression patterns from the TissueAtlas database revealed exceptionally high *miR-29* levels in the brain. Notably, *miR-29a* and *miR-29b* emerged as predominant members of the *miR-29* family in tissues, comprising over 90% of total *miR-29* levels (Figure 2A). RT-qPCR confirmed that the *miR-29* family was enriched in the hypothalamus compared to the testis and epididymis (Figure 2B–D). In addition, widespread expression of *miR-29a* and *-29b* was observed in the hypothalamus (Figure 2E). Next, we assessed the expression levels of *miR-29* family in the hypothalamus of WT, *miR-29ab1*^−/−^, and *miR-29b2c^−/−^* mice. Intriguingly, a complete loss of *miR-29a* expression in the hypothalamus of *miR-29ab1*^−/−^ mice, whereas *miR-29b* expression levels were reduced by 65.79% can be observed (Figure 2F). However, the expression of *miR-29b* showed no significant change, while the expression level of *miR-29c* decreased by 77.44% in the hypothalamus of *miR-29b2c*^−/−^ mice (Figure S4). These results suggest that *miR-29ab1* is the major gene cluster responsible for *miR-29* family functions. 

Circulating testosterone regulates aggressive and mating behaviors by activating AR. In the brain, cells that express aromatase convert serum testosterone into estrogen, which locally controls aggressive behavior [16,37] (Figure 2G). Although the expression levels of CYP19 (Cytochrome P450 19, known as Aromatase) remained unchanged, a significant reduction in AR expression levels was observed in *miR-29ab1*^−/−^ mice, together with a significant increase in ERα expression (Figure 2H–K). Furthermore, a significant decrease in the number of AR^+^ cells (−67.38%) and a significant increase in the number of ER^+^ cells (+85.35%) in the VMH of *miR-29ab1*^−/−^ mice were observed (Figure 2L–O). Overall, results show that *miR-29ab1* is the predominant cluster in the hypothalamus, suggesting that deletion of *miR-29ab1* may impair aggressive and mating behaviors by affecting hypothalamic AR or ERα expression.

### 2.3. Deficiency of miR-29ab1 Leads to Hypothalamic Inflammation and Disrupts the Expression of Genes Linked to Male-Typical Behaviors

**Figure 3:**
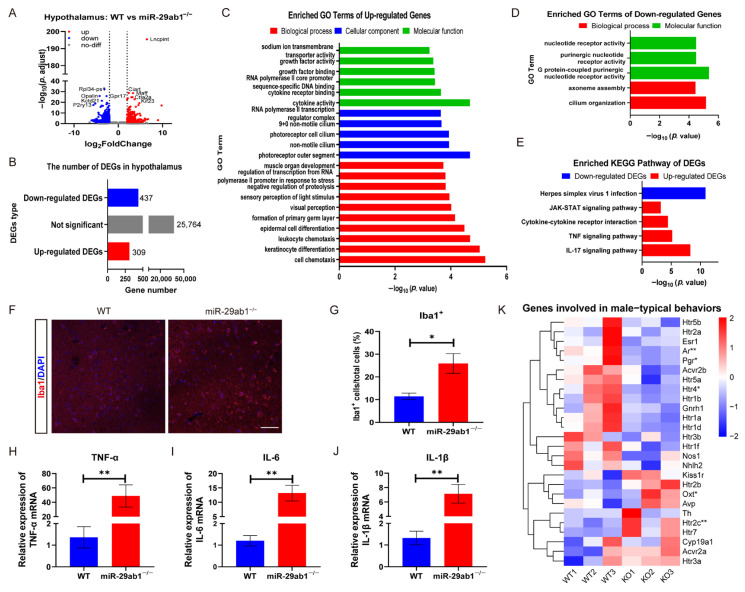
Transcriptomic characteristics of the hypothalamus of WT and *miR-29ab1*^−/−^ mice (*n* = 3). (**A**) Volcano plot showing upregulated (red), downregulated (blue), and non-significantly changed (grey) DEGs (adjust *p*-value < 0.05, |log_2_FoldChange| > 2). (**B**) Number of upregulated, downregulated, and nonsignificant DEGs. (**C**,**D**) GO analysis of upregulated (**C**) and downregulated DEGs (**D**). (**E**) KEGG analysis of up-regulated (red) and down-regulated (blue) DEGs. (**F**) Representative immunofluorescence images showing Iba1^+^ microglia in the hypothalamus of WT and *miR-29ab1*^−/−^ mice. Scale bars, 50 μm. (**G**) The number of Iba1^+^ cells in the hypothalamus of WT and *miR-29ab1*^−/−^ mice (*n* = 6). (**H**–**J**) RT-qPCR analysis of three pro-inflammatory cytokines, TNF-α (**H**), IL-6 (**I**), and IL-1β (**J**) in WT and *miR-29ab1*^−/−^ mice (*n* = 6). (**K**) Heatmap of genes involved in male-typical behaviors (* adjust *p*-value < 0.05, ** adjust *p*-value < 0.01 for heatmap). DEGs, differentially expressed genes; GO, Gene Ontology; KEGG, Kyoto encyclopedia of genes and genomes; KO, *miR-29ab1*^−/−^ mice. * *p* < 0.05, ** *p* < 0.01.

To further explore the function of *miR-29ab1* in the hypothalamus, we next conducted RNA-Seq to analyze transcriptomic data. Firstly, based on the selection criteria, a total of 746 DEGs were identified in the hypothalamus, consisting of 309 up-regulated and 437 down-regulated DEGs in *miR-29ab1*^−/−^ mice relative to WT mice (Figure 3A,B). Specifically, the GO functions of up-regulated DEGs in *miR-29ab1*^−/−^ mice were found to be primarily associated with cell chemotaxis, differentiation, cilium, cytokine activity, and RNA polymerase II (Figure 3C, Table S4). The GO functions of down-regulated DEGs included cilium organization, axoneme assembly, and receptor activity (Figure 3D, Table S5). Notably, the up-regulated KEGG-enriched pathways were associated with four inflammatory signaling pathways, including IL-17, TNF, cytokine-cytokine receptor interaction, and JAK-STAT (Figure 3E, Table S6). Enrichment of KEGG pathway analysis revealed that down-regulated DEGs were associated with herpes simplex virus 1 infection (Figure 3E, Table S7). Secondly, immunofluorescence staining showed that the number of Iba1^+^ microglia was significantly higher in the hypothalamus of *miR-29ab1*^−/−^ mice than in those of WT mice (Figure 3F,G). Furthermore, the proinflammatory cytokines were significantly higher in the hypothalamus of *miR-29ab1*^−/−^ mice than in WT mice, including TNF-α, IL-6 and IL-1β (Figure 3H–J). Additionally, we also identified a significant reduction in the expression of various genes associated with male-typical behaviors in *miR-29ab1*^−/−^ mice, including *Ar*, *Pgr*, and *Htr4*, while *Oxt* and *Htr2c* were significantly increased (Figure 3K). These findings demonstrate that deletion of hypothalamic *miR-29ab1* may contribute to inflammation and influence the expression of genes linked to male-typical behaviors.

### 2.4. miR-29ab1 Directly Regulates Zfp36 in the Hypothalamus

**Figure 4:**
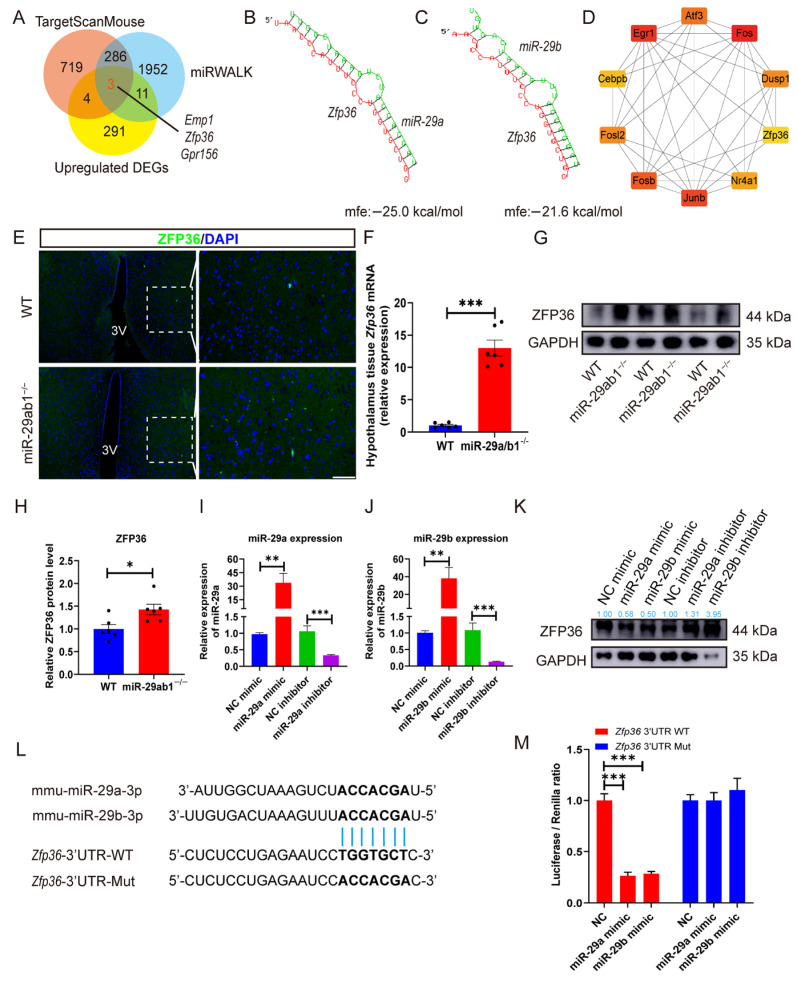
*Zfp36* is the target gene of *miR-29a/b*, and highly expressed in the hypothalamus of *miR-29ab1*^−/−^ mice. (**A**) Venn diagram of overlapping genes among the upregulated DEGs in the hypothalamus, as identified from TargetScanMouse and miRWalk datasets. (**B**,**C**) Minimum free energy (mfe) of the *miR-29a* (**B**) and *miR-29b* (**C**) seed sequence and the 3′-UTR of the predicted target genes from the RNAhybrid dataset. The green strand indicates the predicted miRNA strand (*miR-29a* or *miR-29b*), while the red strand indicates the target (*Zfp36*) in the mouse genome. (**D**) The top 10 hub genes among the upregulated DEGs in the hypothalamus and calculated by MCC method. Colors indicate the score of the genes. The red color denotes the highest degree, while the yellow color signifies the lowest degree. The linkage represents an interaction between the two proteins. (**E**) Representative immunofluorescence images showing ZFP36 staining in the hypothalamus of WT and *miR-29ab1*^−/−^ mice. The image on the right is a magnified image of the white dashed box on the left. Scale bars, 50 μm. (**F**) RT-qPCR analysis of *Zfp36* mRNA levels in WT and *miR-29ab1*^−/−^ mice, and each dot represents a sample (*n* = 6). (**G**) Representative western blot showing ZFP36 protein expression levels in the hypothalamus of *miR-29ab1*^−/−^ mice relative to WT (*n* = 3). GAPDH served as the loading control. (**H**) Quantification of western blot data. Relative ZFP36 protein expression levels in the hypothalamus were normalized to GAPDH (*n* = 6). (**I**,**J**) Relative *miR-29a* (**I**) and *miR-29b* (**J**) expression levels in GT1-7 cells treated with NC mimic/inhibitor, *miR-29a/b* mimic/inhibitor (*n* = 6). (**K**) Representative western blot showing ZFP36 protein expression levels in GT1-7 cells treated with NC mimic/inhibitor, or *miR-29a/b* mimic/inhibitor (*n* = 3). GAPDH served as a loading control. The blue number shows the ratio of average intensity of ZFP36/GAPDH. (**L**) The predicted binding locations for *miR-29a-3p* and *miR-29b-3p* on the 3′-UTR of *Zfp36*. (**M**) Overexpression of *miR-29a/b* led to reduced expression of luciferase reporter gene, dependent on the *Zfp36* 3′-UTR. GT1-7 cells were transferred with luciferase reporter genes regulated by either WT or Mut *Zfp36* 3′-UTR, along with NC RNA mimic or *miR-29a/b* mimic duplexes (*n* = 6/group). 3V, third ventricle; mfe, minimum free energy; MCC, Matthews Correlation Coefficient; ZFP36, zinc finger protein 36; DAPI, 4′6-diamidino-2-phenylindole. * *p* < 0.05, ** *p* < 0.01, *** *p* < 0.001.

To further seek out potential direct targets of *miR-29* in the hypothalamus, we performed bioinformatics analysis by intersecting up-regulated DEGs with known *miR-29* targets from the TargetScanMouse and the miRWalk databases. Three candidate genes (*Emp1*, *Zfp36*, and *Gpr156*) were identified as capable of binding to the *miR-29* seed sequence (Figure 4A, Table S8). Next, we used the RNAhybrid database to analyze the minimum free energy (mfe) of the *miR-29a/b* seed sequences and 3′-UTRs of target genes. No binding sites were predicted for *Emp1* and *Gpr156*. However, the mfe values of the *miR-29a* and *miR-29b* seed sequences and 3′-UTRs of *Zfp36* were found to be −25.0 kcal/mol and −21.6 kcal/mol, respectively (Figure 4B,C). Larger absolute mfe values correlate with more stable binding site structures [36]. We predicted that both *miR-29a* and *miR-29b* could potentially bind to *Zfp36*. Notably, ten hub genes among the 309 up-regulated DEGs were identified by the Matthews Correlation Coefficient (MCC) method, and *Zfp36* was identified as one of the hub genes (Figure 4D, Table S9).

Immunofluorescence staining of our in vivo samples revealed that Zfp36^+^ cells were more enriched in the VMH of *miR-29ab1*^−/−^ mice than WT mice (Figure 4E). Similarly, RT-qPCR analysis confirmed that deletion of *miR-29ab1* led to an approximately 11.51-fold increase in *Zfp36* expression (Figure 4F). Furthermore, ZFP36 protein expression levels were found to be significantly elevated in the hypothalamic tissue of *miR-29ab1*^−/−^ mice compared to WT mice (Figure 4G,H). Next, we transfected GT1-7 cells with *miR-29a/b* mimics or inhibitors. RT-qPCR data revealed that transfection with *miR-29a/b* mimics significantly increased *miR-29a/b* expression levels, while treatment with *miR-29a/b* inhibitors significantly decreased *miR-29a/b* expression levels (Figure 4I,J). Western blot analysis confirmed that endogenous ZFP36 is regulated by *miR-29a/b* (Figure 4K). Next, we cloned the partial mouse *Zfp36* 3′-UTR containing the predicted *miR-29a* target site into a dual-luciferase reporter plasmid, then cotransfected GT1-7 cells with *miR-29a-3p* or *miR-29b-3p* mimics and WT or Mut reporter plasmids. We found that cotransfection of the *miR-29a-3p* mimic or *miR-29b-3p* mimic with WT *Zfp36* led to a significant decrease in fluorescence, which was not observed in the Mut *Zfp36* group (Figure 4L,M), indicating that *Zfp36* is the target of *miR-29a/b*.

### 2.5. Zfp36 Inhibits AR Expression by Targeting RelB

**Figure 5:**
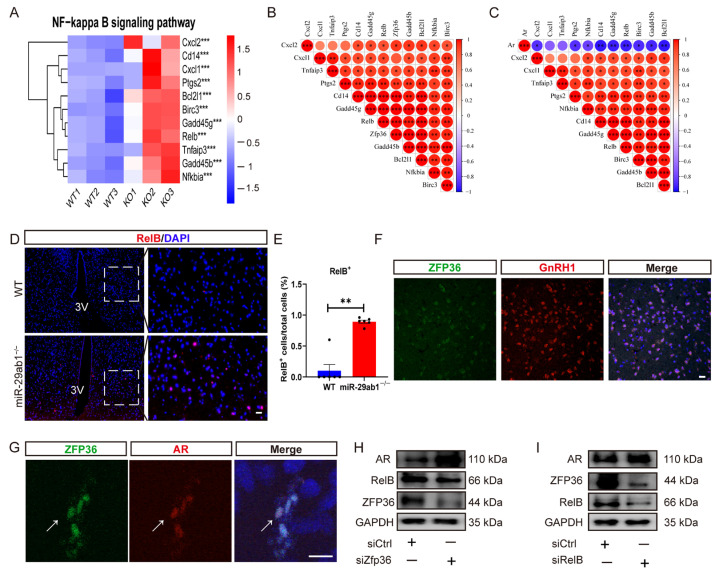
*Zfp36* targets RelB to inhibit AR expression. (**A**) Heatmap of 11 genes activated in the NF-κB signaling pathway. (**B**) Correlation analysis of *Zfp36* and 11 genes involved in the NF-κB signaling pathway. (**C**) Correlation analysis of *Ar* and 11 genes involved in the NF-κB signaling pathway. (**D**) Representative immunofluorescence images showing RelB staining in the hypothalamus of WT and *miR-29ab1*^−/−^ mice. Scale bar, 20 μm. (**E**) The number of RelB^+^ cells in the hypothalamus of WT and *miR-29ab1*^−/−^ mice. Each dot represents a sample (*n* = 6). (**F**) Representative images showing the costaining of ZFP36 and GnRH1 in the hypothalamus. Scale bar, 20 μm. (**G**) Representative images showing costaining of ZFP36 and AR in GT1-7 cells. White arrows indicate co-localized cells. Scale bar, 20 μm. (**H**,**I**) Representative images of western blot showing AR, RelB, and ZFP36 protein expression after treatment of GT1-7 cells with siZfp36 (**H**) or siRelB (**I**) for 48 h. siControl samples received scrambled siRNA (siCtrl); 3V, third ventricle; ZFP36, zinc finger protein 36; GnRH1, gonadotropin-releasing hormone 1; AR, androgen receptor; DAPI, 4′6-diamidino-2-phenylindole. * *p* < 0.05, ** *p* < 0.01, *** *p* < 0.001.

Next, we sought to determine how *Zfp36* regulates AR. Analysis of transcriptomic data revealed activation of the NF-κB signaling pathway in the hypothalamus of *miR-29ab1*^−/−^ mice compared to WT mice (Figure 5A). Interestingly, *Zfp36* expression was found to be positively correlated with the NF-κB signaling pathway, specifically *Cd14*, *Gadd45g*, *Relb*, and *Bcl2l1* gene expression levels (Figure 5B). In contrast, *Ar* was negatively correlated with these four NF-κB-related genes (Figure 5C). Since transcription factors are critical for regulating gene expression, we then investigated the role of *Relb* in mediating the regulation of *Ar* by *Zfp36*.

The RelB^+^ cell population was significantly increased in the entire hypothalamus of *miR-29ab1*^−/−^ mice relative to WT mice, including the region of VMH (Figure 5D,E). Moreover, costaining analysis revealed the colocalization of ZFP36 and GnRH1 in the hypothalamus (Figure 5F). The GT1-7 cell line originates from GnRH-producing neurons in the hypothalamus, and AR is expressed in primary GnRH neurons [37,38]. Immunofluorescence staining revealed that ZFP36 and AR colocalize within GT1-7 cells (Figure 5G), suggesting an interaction between ZFP36 and AR. Subsequently, following knockdown of ZFP36 in GT1-7 cells using a siRNA specific for Zfp36 (siZfp36), we observed an increase in AR expression and a decrease in RelB expression (Figure 5H). In accordance with these findings, treatment of GT1-7 cells with siRelB resulted in a downregulation of ZFP36 expression together with a concomitant increase in AR expression (Figure 5I). Overall, these findings suggest that *Zfp36* regulates AR expression via *RelB*.

## 3. Discussion

This study clarified the crucial role of the *miR-29ab1* cluster in regulating reproduction and male-typical behaviors through regulation of AR expression in the hypothalamus, particularly the VMH. Past studies have revealed that genetic deletion of *miR-29ab1* leads to sterility and causes ovulation disorders in female mice [33,34]. These effects were sex-dependent in brain-specific deficiency of *miR-29*, resulting in hyperfertility in females and subfertility in males [36]. Our study demonstrates that the *miR-29ab1* cluster is the predominant component of the *miR-29* family, which is highly enriched in the hypothalamus. We show that ablation of *miR-29ab1* leads to reduced male-typical behaviors in male mice, which can be attributed to an imbalance in homeostasis maintained by the hypothalamus, as evidenced by altered expression of multiple genes related to male-typical behaviors, including *Ar* and *miR-29* target genes (*Zfp36*). Furthermore, data indicate that *miR-29ab1* inhibits AR expression by targeting *Zfp36*. Notably, inhibition of *RelB* restores AR expression, suggesting a regulatory pathway involving the *miR-29ab1/Zfp36/AR* axis of the hypothalamus in male-typical behaviors.

miRNAs are key regulators across a wide array of biological functions and have been implicated in influencing diverse behaviors. The *miR-29* family is widely expressed in the brain, particularly in the cortex, hippocampus, and cerebellum, highlighting its importance in neural regulation [30]. *miR-29* has been implicated in processes such as neural cell death and atrophy [30,38]. Interfering with just one miRNA can hinder its capacity to regulate a complex physiological function *in vivo*, as most miRNAs achieve their effects by targeting multiple mRNAs within the same or interconnected pathway. Recent studies using cellular or tissue knockout systems have shown that *miR-29* regulates the onset of mammalian puberty and reproduction by modulating luteinizing hormone secretion, ovulation, or *Gnrh1* expression [34,35,39]. These effects are mediated through direct targeting of genes such as *Ptx3* and *Tbx21*.

Some studies have shown that global deletion of *miR-29* in mice leads to early mortality [40]. Moreover, significant phenotypic differences have been observed in mice lacking the *miR-29ab1* or the *miR-29b2c* cluster. In comparison to *miR-29b2c*^−/−^ mice, *miR-29ab1*^−/−^ mice exhibit numerous phenotypes, including sterility [33]. Although no abnormalities in the weights of the testes and seminal vesicles have been reported in *miR-29ab1*^−/−^ males [34], males with brain-specific knockdown of *miR-29* have been shown to exhibit subfertility [38]. In contrast to the impaired aggressive and mating behavior of *miR-29ab1*^−/−^ mice, *miR-29b2c*^−/−^ mice only showed reduced aggression. This difference in behavioral phenotypes may be attributed to the different expression patterns of *miR-29b2c.* On one hand, *miR-29c* is expressed at lower levels in the brain compared to *miR-29a* and *miR-29b*. On the other hand, the expression of *miR-29b* is strongly correlated with *miR-29a* but not with *miR-29c* [33]. Thus, our objective is to ascertain whether *miR-29ab1* plays a role in mediating male-typical reproductive behaviors, including aggression and mating. Our findings reveal that, while male mice exhibit typical spontaneous mobility and reproductive tissue health, the deletion of *miR-29ab1* leads to the absence of both aggressive and mating behaviors in male mice. 

Male aggressive and mating behaviors are regulated by neural circuits and endocrine systems and are dependent on testosterone. Testosterone in the bloodstream activates AR. In the brain, it is also converted into estrogen by aromatase, leading to the activation of ERα-mediated signaling [12]. A sharp drop in testosterone due to castration has been shown to eliminate male-typical behaviors in mice [17]. Moreover, AR-null mutations in males reduce male-typical behaviors [12,15]. Several genes regulate male-typical behaviors, such as *Oxt*, *Avp*, *Calca*, *Th*, *Tph*, *5-HT1A*, *5-HT1B*, *nNOS*, *B3gnt1*, and *Sytl4* [13,15,41]. Consistent with the known role of these genes, loss of *miR-29ab1* alters the expression of several genes in the hypothalamus, including *Ar*, *Pgr*, *Htr4*, *Oxt*, and *Htr2c*. Abnormal gene expression caused by *miR-29ab1* deficiency is related to the activation of hypothalamic inflammation and the expression of diverse target genes. In the prostate, reduced AR signaling in prostatic luminal cells promotes immune cell infiltration and increases inflammatory markers [42], suggesting an association between diminished AR expression and increased inflammation. However, whether reduced AR expression in the brain activates inflammation remains unknown.

*MiR-29* modulates inflammation by directly targeting inflammatory genes, such as TNF receptor 1 (*Tnfrsf1a*) and interferon- γ (*IFN-γ*) [43,44]. In the present study, multiple inflammation-related signaling pathways have been activated in the hypothalamus that lacks *miR-29ab1*, including the JAK-STAT, cytokine-cytokine receptor interaction, TNF, IL-17, and NF-κB signaling pathways. Meanwhile, the RNA-binding protein *Zfp36*, associated with immunomodulation, was identified as a direct target of *miR-29a/b*. Previous studies have shown that *Zfp36* regulates mRNA stability by binding to particular sequences in the 3′-UTR of target mRNAs. These sequences are typically characterized by AU-rich elements (AREs) [45,46]. Mice with disrupted *Zfp36* expression have been shown to develop severe arthritis, autoimmunity, cachexia, dermatitis, and myeloid hyperplasia, due to the overexpression of TNF-α [47,48]. The inflammatory response can be attenuated by overexpressing *Zfp36* [49]. Interestingly, the binding of inducible *Zfp36* to AREs to mediate the degradation of inflammatory factors occurs not only in immune cells, but also in nonimmune cells. For example, overexpression of *Zfp36* in HeLa cells led to the upregulation of genes, including RelB, a key member of the NF-κB signaling pathway [46]. In this study, ZFP36 and AR were found to colocalize in GT1-7 cells, suggesting a reciprocal relationship between these two proteins. Further inhibition of *Zfp36* or *RelB* in GT1-7 cells resulted in an increase in AR expression, suggesting a potential feedback mechanism where the suppression of NF-κB signaling indirectly affects the stability and expression of mRNAs targeted by *Zfp36*. These findings reveal an unexpected level of control over male-typical behaviors by *miR-29ab1* and indicate a more profound connection of inflammation, sex steroid receptors, and control of male-typical behaviors. The suppression of AR expression via NF-κB activation could represent a feedback mechanism in response to inflammatory states, potentially influencing reproductive capacity and behavior under stress or disease conditions.

The potential role of regulatory networks involving *miR-29ab1*, *Zfp36*, and AR in humans is of interest. This is partly due to the conserved expression of the *miR-29* family in humans [31], as well as the critical role of AR in regulating human libido and sexual desire [50]. While this study has elucidated the pivotal role of the *miR-29ab1* cluster in regulating reproduction and male-typical behaviors by affecting AR expression in the hypothalamus, it has several limitations. First, although we have shown the impact of *miR-29ab1* on AR and *Zfp36* expression, our study did not include a comprehensive analysis of all potential *miR-29* targets, such as *Emp1* and *Gpr156*, which could also contribute to the observed phenotypes. In addition, our study did not differentiate between the effects of *miR-29a* and *miR-29b*, potentially obscuring unique contributions of each miRNA within the cluster to the regulation of male-typical behaviors. The reliance on knockout models also limits our understanding of the dynamic regulation of these miRNAs under physiological conditions. Future studies should use conditional gene knockout mouse models to elucidate the roles of individual miRNAs and integrate comprehensive multi-omics approaches to better understand the interactions governed by *miR-29ab1* in the regulation of male-typical behaviors and reproduction.

## 4. Materials and Methods

### 4.1. Generation of Mouse Models

Two mouse models, *miR-29ab1* knockout (*miR-29ab1*^−/−^) and *miR-29b2c* knockout (*miR-29b2c^−/−^*) mice, were generously provided by Dr. Zhengquan Yu (Beijing, China) [40]. The *miR-29ab1* heterozygote (*miR-29ab1*^+/−^) strain was obtained through in vitro fertilization using sperm from *miR-29ab1*^−/−^ male mice and eggs from WT female mice, which was performed by Model Organisms (Shanghai, China). *MiR-29ab1*^+/−^ strain was crossed with itself to obtain *miR-29ab1*^+/+^ (WT) and *miR-29ab1*^−/−^ mice. The primers employed for genotyping derived from tail biopsies are detailed in Table S1. Unless specified otherwise, all animals included in this study were male and aged between 2 and3 months. The mice were housed in a pathogen-free animal facility under controlled conditions, including a 12:12 light/dark cycle with lights off at 20:00, and a stable environment maintained at 22 ± 2 °C and 45% ± 10% humidity. This research was approved by the Institutional Animal Care and Use Committee of China Agricultural University, in accordance with the guidelines for the care and use of laboratory animals (approval number: AW51203202-5-2).

### 4.2. Cells

Murine hypothalamic GT1-7 cells were obtained from Jinyuan Biotechnology Co., Ltd. (Shanghai, China) and cultured in Dulbecco’s Modified Eagle Medium (DMEM, Gibco, Grand Island, NY, USA). This medium was enriched with 8% fetal bovine serum (Gibco) and 2% horse serum (Gibco). In this case, 1 × penicillin-streptomycin (Beyotime, Shanghai, China) was included. The cells were then kept in a humidified incubator at 37 °C with 5% CO_2_.

### 4.3. Cell Transfection

MiR-29a/b mimics, inhibitors, and corresponding negative control mimic and inhibitor were synthesized by GenePharma (Shanghai, China). The sequences of the sense strands are listed in Table S2. To achieve overexpression or inhibition of *miR-29a* or *miR-29b*, GT1-7 cells were seeded at a density of 2 × 10^5^ cells/well in 6-well plates and cultured at 37 °C until they reached 60–70% confluence. For *miR-29a/b* overexpression or inhibition, cells were treated with Opti-MEM (Gibco) and transfected with 40 nmol·L^−1^ *miR-29a/b* mimics or inhibitors together with Lipofectamine 2000 reagent (Invitrogen, Carlsbad, CA, USA) for 6 h.

For knocking down *Zfp36* or *RelB*, cells were transfected with 50 nmol·L^−1^ small interfering RNA (siRNA)(siZfp36 or siRelB, GenePharma, China) in Opti-MEM and 1 μL Lipofectamine 2000 reagent. The two solutions were gently mixed and incubated for 20 min at room temperature to form the transfection vector mixture. RNA and protein extractions were performed 48 h later. The siZfp36 are listed with the following sequences: 5′-UGAAGUGGCAUCGAGAGCCTT-3′, and the siRelB are listed with the following sequences: 5′-UUGGGAAACAUGUUGCUGCTT-3′.

### 4.4. Fertility Assessment

Male mice were housed with wild-type (WT) female mice in a 1:4 ratio. The presence of a vaginal plug was checked each morning over a period of 21 consecutive days to evaluate the success of mating. Pregnant mice were isolated, and the number of litter size was documented. Mice that did not become pregnant were allowed to continue mating. Male mice that failed to impregnate any of the four females within one-month period were categorized as infertile.

### 4.5. Behavioral Tests

#### 4.5.1. Open Field Test

Autonomous exploration tests were conducted for 10 min and monitored with Supermaze software (version 3.3.0.0, Xinruan, Shanghai, Beijing). The trajectory, total locomotor activity, distance, and fecal boli count were systematically recorded (*n* = 10/group).

#### 4.5.2. Aggressive Behavior

Male-male aggression was evaluated using the resident-intruder paradigm [15]. Briefly, resident mice were singly isolated at 6–8 weeks of age, and their bedding remained unchanged during the last seven days. Testing was initiated with the introduction of an unfamiliar, sexually inexperienced, group-housed adult WT male mouse (intruder) into the home cage of the resident mouse (resident), and the testing lasted 15 min. Recorded parameters included the number of resident mice displaying aggression, attack duration, attack frequency, and latency to first attack (*n* = 10/group).

#### 4.5.3. Mating Behavior

One week after the aggressive behavior test, sexually inexperienced mice participated in a mating assay. Testing began once an estrous WT female mouse was introduced into the home cage of the test mouse. Males initiate a series of sex-specific stereotypical motor outputs, including mounting, intromission, and ejaculation [51]. The mating behavior of male mice starts with mounting attempts, which were quantified according to the percentage of mice displaying mounting behavior, total number and duration of mounts, and first mount latency (*n* = 13/group).

### 4.6. Histological Analysis

Mice were killed by isoflurane inhalation and transcardially perfused with 0.9% saline, followed by 4% paraformaldehyde. The brain was harvested and fixed in 4% paraformaldehyde, while testis and epididymis were fixed for 24 h in Bouin’s solution (Servicebio, Wuhan, China). Testis, epididymis, and brain sections were embedded in paraffin by standard procedures. Then, tissues were sectioned (5 μm) and performed hematoxylin-eosin (H&E) staining. The testis and epididymis were assessed by measuring the outer and inner seminiferous tubule diameter, and thickness of epithelial epididymis as described previously (*n* = 10/group, three replicates) [52]. Both indicators were calculated from five tubules per section using ImageJ software (Version 1.52a, NIH, Bethesda, MD, USA).

### 4.7. Sperm Motility

Sperm concentration and motility were analyzed by computer-aided sperm analysis (CASA) as described previously [53]. Briefly, a small incision was created in the cauda epididymis using sterile microscissors. Spermatozoa were extracted from the cauda epididymis and placed in 1 mL phosphate-buffered saline (PBS) for 15 min at 37 °C in an atmosphere of 5% CO_2_. The suspended sperm (10 μL) from each sample (*n* = 8/group, two replicates) was placed dropwise onto a specialized slide for sperm motility testing. The slide was covered, then placed on a microscope set to 37 °C. Sperm motility indices were collected and recorded using the CASA system (Version 12 CEROS, Hamilton Thorne Research, Beverly, MA, USA).

### 4.8. Quantitative Reverse Transcription Polymerase Chain Reaction (RT-qPCR)

RNA was isolated from the lung, testis, epididymis, hypothalamus or cells using RNA-easy Isolation Reagent (Vazyme, Nanjing, China), and was converted to complementary DNA (cDNA) according to the protocol of HiScript III All-in-one RT SuperMix Perfect (Vazyme). Results were normalized by *U6* for *miR-29* and *Gapdh* for mRNA.

For *miR-29*, RNA was isolated and converted using Moloney Murine Leukemia Virus Transcriptase (Promega Madison, WI, USA), deoxynucleotide triphosphate (Sigma, Darmstadt, Germany), and RNasin Ribonuclease Inhibitors (Promega) according to the previous method [32]. For mRNA, cDNA was synthesized using HiScript III All-in-one RT SuperMix (Vazyme). RT-qPCR was conducted with ChamQ Universal SYBR qPCR Master Mix (Vazyme). The primer sequences are displayed in Table S3.

### 4.9. Western Blot

Protein was isolated from hypothalamus and cells using RIPA buffer (Beyotime). Equal quantities of protein were resolved by SDS-PAGE, subsequently transferred to polyvinylidene fluoride membranes, and incubated overnight at 4 °C with primary antibodies. Then, blots were treated with the horseradish peroxidase (HRP)-conjugated secondary antibodies corresponding to the primaries. Immunoreactive bands were developed with Robust Enhanced Chemiluminescent (ECL) HRP solution (Affinbody, Wuhan, China). The primary antibodies were as follows: AR (1:1000, Abclonal, Wuhan, China), cytochrome p450 aromatase (CYP19; 1:1000, Santa Cruz, Dallas, TX, USA), estrogen receptor alpha (ERα; 1:1000, Affinity, Changzhou, China), ZFP36 (1:1000, Santa Cruz), RelB (1:1000, Abclonal), and GAPDH (1:10,000, Proteintech, Rosemont, IL, USA). Goat anti-rabbit (1:5000, Beyotime) or goat anti-mouse (1:5000, Beyotime) secondary antibodies were used. 

### 4.10. Fluorescence In Situ Hybridization (FISH)

The fresh brain samples were fixed and then embedded in paraffin. Subsequently, tissues were sectioned into 5 μm-thick coronal sections using the Mouse Brain Atlas as reference [54]. After slicing and dewaxing, sections were boiled in retrieval solution for 15 min, then incubated with proteinase K (20 μg/mL) for 30 min at 37 °C. Sections were washed with PBS, then incubated with prehybridization solution for 30 min at 37 °C. Next, the probe-containing hybridization solution was added, and samples were hybridized overnight at 37 °C. Samples were rinsed with saline-sodium citrate (SSC) at gradients of 2×, 1×, and 0.5× SSC at room temperature for 10 min. Nuclei staining was performed with 4′,6-diamidino-2-phenylindole (DAPI) (Servicebio) for 8 min. Then, the slides were mounted and visualized with CaseViewer (version 2.4, Budapest, Hungary).

### 4.11. Immunofluorescence and Immunochemistry

The hypothalamic sections were subjected to deparaffinization and hydration, followed by immersion in sodium citrate antigen retrieval solution (Servicebio) and microwave boiling. Sections were treated with primary antibodies overnight at 4 °C, followed by incubation with horseradish peroxidase (HRP)-conjugated (ZSGB-BIO, Beijing, China) or florescence-labeled secondary antibodies. The following primary antibodies were as follows: Iba1 (1:500, Proteintech), GnRH1 (1:1000, Abclonal), AR (1:1000, Abclonal), ZFP36 (1:500, Santa Cruz), ERα (1:1000, Affinity, Jiangsu, China), and RelB (1:1000, Abclonal). After incubation with diaminobenzidine and hematoxylin for immunohistochemistry or DAPI, stained samples were visualized by CaseViewer (Budapest, Hungary) or a digital pathology scanner (KFBIO, version 1.7.1.1, Yuyao, China).

### 4.12. RNA-Sequencing Analysis

RNA sequencing analysis of hypothalamus samples was carried out by Novogene (Beijing, China). Library quality was evaluated using Agilent Bioanalyzer 2100 system, and libraries were prepared using the Illumina Novaseq platform, generating 150 bp paired-end reads. The mapped reads were assembled by StringTie (v1.3.3b) and converted to FPKM (fragments per kilobase of transcript sequence per million base pairs sequenced) by featureCounts (v1.5.0-p3). Differentially expressed genes (DEGs) of WT and *miR-29ab1*^−/−^ mice were selected using R-package DESeq2 (version 1.40.2) with the following criteria: adjust *p*-value < 0.05 and |log_2_(FoldChange)| > 2. Functional analysis of genes using gene ontology (GO) annotation and Kyoto Encyclopedia of Genes and Genomes (KEGG) pathway enrichment were conducted using R software (version 4.3.1).

### 4.13. Dual-Luciferase Assay

The target prediction database TargetScanMouse (version 8.0) indicated that there were binding sites between *miR-29* and the 3′-UTR of *Zfp36*, and it was examined in GT1-7 cells. Cells were seeded one day prior to transfection. WT and mutant (Mut) Zfp36-3′-UTRs were cloned into GP-miRGLO plasmids (GenePharma). Cells were cotransfected with *miR-29a* mimic, *-29b* mimic, or its negative control mimic. After 24 h, the activities of Firefly luciferase were measured using the Dual-luciferase reporter assay system (Vazyme), with results normalized to the relative light units of Renilla according to the manufacturer’s instructions.

### 4.14. Bioinformatics Analysis

The online database TissueAtlas was used for *miR-29* expression levels in tissue samples (https://ccb-web.cs.uni-saarland.de/tissueatlas2/, accessed on 16 November 2024) [55]. The putative targeting genes of *miR-29* were identified based on TargetScanMouse and miRWALK (version 3) databases. Another online database STRING (version 12.0) was used to construct a protein-protein interaction (PPI) network with the minimum required interaction score set at 0.700. The hub genes were identified through Cytoscape software (version 3.10.1). The minimum free energy (mfe) between the *miR-29* seed sequence and 3′-UTRs of target genes was analyzed using RNAhybrid (version 2.2.2).

### 4.15. Statistical Analysis

All results are reported as the mean ± standard error of the mean, except the pregnant rate and percentage of exhibiting male-typical behaviors. The percentages of animals exhibiting behavior were carried out with a chi-square test. The remaining data underwent a normality check using Shapiro–Wilk test. For comparing normally distributed data, the two-independent samples *t* test was utilized, while the Mann-Whitney *U* test was employed for intergroup comparison of non-normally distributed data. To assess differences among groups, a one-way analysis of variance (ANOVA) followed by a Bonferroni’s post-hoc test was conducted. *p*-value lower than 0.05 was considered significant. SPSS software (version 26.0, SPSS Inc., Chicago, IL, USA) was used to conduct all statistical analyses. GraphPad Prism software (version 8.0.2, GraphPad Software Inc., San Diego, CA, USA) was carried out for graph plotting. Image J software (version 1.54f, National Institutes of Health, USA) was used for the quantification of blot and image.

## 5. Conclusions

In summary, our findings further our understanding of the *miR-29ab1* cluster in regulating reproduction and male-typical behaviors through its effects on AR expression in the hypothalamus. Our study establishes a link between the disruption of hypothalamic homeostasis and the alteration of gene expression patterns, including key targets such as *Zfp36*. Furthermore, our findings underscore the critical role of *miR-29ab1* in modulating AR expression through its impacts on the NF-κB signaling pathway, enhancing our understanding of the neural mechanisms controlling reproductive behaviors.

## Figures and Tables

**Table 1:** Sperm mobility of WT and *miR-29ab1*^−/−^ mice under physiological conditions (*n* = 8).

Item	WT	miR-29ab1^−/−^	*p* Value
Total concentration (M/mL)	541.30 ± 78.78	480.15 ± 38.74	0.497
Motile/%	27.75 ± 2.66	35.13 ± 3.88	0.139
Average path velocity (μm/s)	14.63 ± 1.49	14.66 ± 1.21	0.985
Straight line velocity (μm/s)	8.48 ± 1.05	8.81 ± 0.72	0.795
Curvilinear velocity (μm/s)	30.70 ± 2.25	31.13 ± 1.42	0.875
Straightness/%	55.13 ± 2.66	57.50 ± 2.56	0.531
Lateral head displacement/μm	2.39 ± 0.16	2.30 ± 0.12	0.663
Beat cross frequency	33.16 ± 2.32	34.33 ± 2.08	0.715
Linearity	30.13 ± 2.48	31.13 ± 2.55	0.783
Elongation ratio	51.00 ± 1.34	49.63 ± 0.46	0.347

## Data Availability

The data in this study are available from the corresponding author upon reasonable request.

## Supplementary Materials

The following supporting information can be downloaded at: https://www.mdpi.com/article/10.3390/ijms252313089/s1.
