# Supplementary material for: The microRNA-29ab1/Zfp36/AR Axis in the Hypothalamus Regulates Male-Typical Behaviors in Mice
Source: Int J Mol Sci. 2024 Dec 5;25(23):13089. doi: 10.3390/ijms252313089 (PMC11642693; doi:10.3390/ijms252313089)
Supplement: Supplementary file 1 [file ijms-25-13089-s001.zip › Supplementary Table 1-3.pdf]

Supplementary Table 1-3:

Table S1. Sequences of primers used for genotyping in *miR-29ab1*$^{-/-}$ and *miR-29b2c*$^{-/-}$ mice.

**Primer name**	**Sequence (5' to 3')**
miR-29ab1-F	TCGGGTGAGGGCTCAGTTACCAT
miR-29ab1-R	CCGTCAAATCTGCAACCCATACAC
miR-29bc-F	TCTACCGACACTATGCATCTTGGGA
miR-29b2c-R	CAGGGACCACTTCTCATTGCCAT

Table S2. Sequences of the sense strands of mimics and inhibitors.

**Primer name**	**Sequence (5' to 3')**
negative control mimic	ACGUGACACGUUCGGAGAATT
miR-29a mimic	ACCGAUUUCAGAUGGUGCUAUU
miR-29b mimic	CACUGAUUUCAAAUGGUGCUAUU
negative control inhibitor	CAGUACUUUUGUGUAGUACAA
miR-29a inhibitor	UAACCGAUUUCAGAUGGUGCUA
miR-29b inhibitor	AACACUGAUUUCAAAUGGUGCUA

Table S3. Sequences of the RT-qPCR primers used in this paper.

**Primer name**	**Sequence (5' to 3')**
miR-29a-RT	CTCAACTGGTGTCGTGGAGTCGGCAATTCAGTTGAGTAACCGAT
miR-29b-RT	CTCAACTGGTGTCGTGGAGTCGGCAATTCAGTTGAGAACACTGA
U6-RT	CGCTTCACGAATTTGCGTGTCAT
RT-miR-29a-F	TCACGTAGCACCATCTGAA
RT-miR-29a -R	GTCAGTCCGTTTGGCCAGTA
RT-miR-29b -F	TCACGTAGCACCATTTGAAA
RT-miR-29b-R	GTCAGTCCGTTTGGCCAGTA
RT-U6-F	CTCGCTTCGGCAGCACA
RT-U6-R	AACGCTTCACGAATTTGCGT
TNFα-F	GACGTGGAACTGGCAGAAGAG
TNFα-R	TTGGTGGTTTGTGAGTGTGAG
IL-6-F	CCAAGAGGTGAGTGCTTCCC
IL-6-R	CTGTTGTTCAGACTCTCTCCCT
IL-1β-F	GCAACTGTTCCTGAACTCAACT
IL-1β-R	ATCTTTTGGGGTCCGTCAACT
Zfp36-F	CCACCTCCTCTCGATACAAGA
Zfp36-R	GCTTGGCGAAGTTCACCCA
Gapdh-F	AGGTCGGTGTGAACGGATTTG
Gapdh-R	TGTAGACCATGTAGTTGAGGTCA
